# Targeting inflammation as a treatment modality for neuropathic pain in spinal cord injury: a randomized clinical trial

**DOI:** 10.1186/s12974-016-0625-4

**Published:** 2016-06-17

**Authors:** David J. Allison, Aysha Thomas, Kayleigh Beaudry, David S. Ditor

**Affiliations:** Department of Kinesiology, Brock University, St Catharines, Ontario L2S 3A1 Canada; Brock-Niagara Centre for Health and Well-being, St Catharines, Ontario L2T 1W4 Canada

**Keywords:** Neuropathic pain, Spinal cord injury, Inflammation, Anti-inflammatory diet

## Abstract

**Background:**

The purpose of the present study was to examine the effectiveness of an anti-inflammatory intervention as a treatment for neuropathic pain following spinal cord injury (SCI).

**Methods:**

This randomized, parallel-group, controlled clinical trial (NCT02099890) examined 20 participants with varying levels and severities of SCI, randomized (3:2) to either a 12-week anti-inflammatory diet, or control group. Outcome measures consisted of self-determined indices of pain as assessed using the neuropathic pain questionnaire (NPQ) and markers of inflammation as assessed by various pro- and anti-inflammatory cytokines, as well as the eicosanoids PGE2 and LTB4.

**Results:**

A significant group × time interaction was found for sensory pain scores (*p* < 0.01). A Mann-Whitney test revealed that the change scores (3-month baseline) were significantly different between groups for IFN-y (*U* = 13.0, *p* = 0.01), IL-1β (*U* = 14.0, *p* = 0.01), and IL-2 (*U* = 12.0, *p* = 0.01). A Friedman test revealed the treatment group had a significant reduction in IFN-y (*x*^2^ = 8.67, *p* = 0.01), IL-1β (*x*^2^ = 17.78, *p* < 0.01), IL-6 (*x*^2^ = 6.17, *p* < 0.05), while the control group showed no significant change in any inflammatory mediator. A stepwise backward elimination multiple regression analysis showed that the change in sensory neuropathic pain was a function of the change in the proinflammatory cytokines IL-2 and IFN-y, as well as the eicosanoid PGE2 (*R* = 0.689, *R*^2^ = 0.474).

**Conclusions:**

Overall, the results of the study demonstrate the efficacy of targeting inflammation as a means of treating neuropathic pain in SCI, with a potential mechanism relating to the reduction in proinflammatory cytokines and PGE2.

**Trial registration:**

ClinicalTrials.gov, NCT02099890

**Electronic supplementary material:**

The online version of this article (doi:10.1186/s12974-016-0625-4) contains supplementary material, which is available to authorized users.

## Background

Damage to the nervous system may result in persistent or permanent changes to nociceptive thresholds resulting in a unique pain state characterized by allodynia and/or hyperalgesia [1]. This form of pain, known as neuropathic pain, effects an estimated 29–75 % of the spinal cord injured (SCI) population [2]. Such a condition can be severely debilitating following SCI and may contribute to inactivity and related metabolic conditions, as well as behavioral disorders such depression, anxiety, and insomnia [3]. Neuropathic pain is notoriously difficult to treat, and no current treatment has been consistently proven as universally effective, predictable, and safe for long-term use [4]. Based on the widespread potential to impact quality of life, it is imperative to gain a better understanding of the underlying mechanisms of neuropathic pain and explore novel treatments.

Traditionally, neuropathic pain has been viewed as an etiology stemming solely from structural damage to the neuron itself. However, it is now well-established that the environment with which the nociceptor interacts is also an important factor [5]. A number of inflammatory mediators are known to reduce the nociceptive threshold, resulting in symptoms of hyperalgesia [5]. Proinflammatory cytokines such as IL-1β (interleukin-1 beta), IFN-y (interferon gamma), IL-6, and TNF-α (tumor necrosis factor-alpha) have been proposed to induce algesic effects by both direct [6] and indirect [7] (prostaglandin-dependent) influences. The inflammatory etiology of neuropathic pain is further supported based on evidence that analgesic effects have been produced following the reduction of proinflammatory cytokine concentrations. This has been demonstrated in animal models following the administration of antibodies against TNF-α [8] as well as in transgenic animal models with impaired IL-1β production [9, 10].

Pharmacological treatment strategies are currently the most heavily relied upon form of neuropathic pain management. Studies have, however, shown mixed results concerning the efficacy, universality, and associated side effects of treatments such as tricyclic antidepressants, SSRI’s, cannabinoids, anticonvulsants, and opioids [4]. This inconsistency across studies may be due, in part, to variability in protocols (e.g., duration, dosage) but may also relate to the varied etiological basis across different participants. A better understanding of the various mechanisms at play and a more advanced mechanism-based classification system will be critical to enhance the specificity and efficacy of current pharmacological treatments. This may contribute to the development of pharmacological treatments which target the underlying mechanisms responsible for neuropathic pain as opposed to focusing solely on symptom relief.

An additive to traditional pharmaceutical treatments may be the implementation of anti-inflammatory interventions. Such strategies would target the common inflammatory mechanisms which underlie various etiologies thereby having the potential to provide a more widely applicable treatment option. Further, if such strategies prove effective, it may be possible to produce such anti-inflammatory effects via simple lifestyle alterations without the use of pharmaceuticals. This may help to reduce the reliance on traditional pharmaceuticals thereby helping to avoid associated side effects. Appropriate dietary alterations including foods and supplements with established anti-inflammatory benefits have been shown to effectively reduce inflammation via a number of mechanisms ranging from altered gene transcription, changes in cell membrane composition, and improved enzyme regulation, as well as improvements in metabolic heath and body composition [11].

Dietary needs, including nutrient requirements and caloric intake, change drastically following SCI and are often not adequately met. Diet is, therefore, likely a substantial contributing factor to the chronic inflammation typically observed following SCI [12, 13]. A dietary intervention consisting of foods and supplements with established anti-inflammatory benefits was therefore utilized in the current study with the intention of reducing inflammation for the purpose of studying the effect on neuropathic pain. It was hypothesized that by reducing chronically elevated levels of inflammatory mediators, corresponding reductions in neuropathic pain would be achieved.

## Methods

### Study design and participants

This study was performed as a component of a larger clinical trial (clinicaltrials.gov identifier: NCT02099890) which included the examination of depression [14], cognitive impairment, and somatic nerve function. Data pertaining to the change in inflammatory mediators has been previously published [14]. The study was a randomized, parallel-group clinical trial. Participant recruitment occurred between September and November 2014. The study intervention was 12 weeks and included testing at baseline, 1 month, and 3 months. Participants with various levels and severities of SCI were recruited for participation in the study. Additional inclusion criteria included (1) over the age of 18, (2) SCI of any level or severity (American Spinal Injury Association A-D), (3) at least 2 years post-injury. Exclusion criteria included (1) any contraindications to supplements provided in the study, (2) unstable dosage of any pain medications, (3) unstable medical condition within 2 weeks prior to intervention, (4) pregnancy, and (5) breastfeeding. Participant characteristics are shown in Table 1. Twenty individuals (10 males, 10 females; age 48.7 ± 13.9 years) with chronic (4–37 years post-injury) SCI (C2-L4; ASIA Impairment Scale (AIS) A-D) were recruited for participation in the study. Twelve participants were randomly allocated to the treatment group and were placed on the 12-week anti-inflammatory diet intervention, while eight were allocated to the control group and received no intervention. This group allocation was selected in order to protect against potential dropouts. Three participants (two from the treatment group and one from the control group) were using medications which could influence pain. These medications included oxycodone and baclofen (used by the two participants of the treatment group) and gabapentin (used by the participant of the control group). Dosages of these medications were, however, maintained throughout the duration of the study. Informed consent was obtained from all participants. The study was registered as a clinical trial (clinicaltrials.gov identifier: NCT02099890) and received ethical approval from the Brock University Research Ethics Board as well as the Natural Health Products Directorate of Canada. All data was collected on-site at Brock University and the Brock-Niagara center for health and well-being.

**Table 1:** Participant characteristics

Participant	Sex	Age	AIS score	Level	Time since injury (years)
Treatment					
1	F	44	D	C5	10
2	M	58	B	T10	4
3	F	62	D	L3	4
4	F	37	A	T3	19
5	M	22	A	C7	5
6	M	67	C	C2	4
7	M	66	D	C5	6
8	F	44	A	C7	9
9	F	65	D	T6	4
10	F	64	D	C3	37
11	M	45	A	T6	28
12	M	37	C	C4	23
Control					
13	F	30	B	C5	6
14	F	63	D	L4	2
15	M	42	A	C5	6
16	F	58	D	C5	33
17	M	59	D	T4	4
18	F	33	A	T1	17
19	M	41	C	C4	22
20	M	36	A	C5	19

*AIS* ASIA (American Spinal Injury Association) Impairment Scale

### Randomization

Randomization was computer generated by the primary investigator and stratified by participant gender and age using permuted blocks of 2 (male/female) and block of 3 (<40, 40–60, >60 years). Randomization was 3:2 to either the anti-inflammatory diet vs control.

### Anti-inflammatory diet intervention

The anti-inflammatory diet intervention focused on the elimination of common food intolerances and inflammation-inducing foods, as well as the introduction of foods and supplements with established anti-inflammatory properties. Examples of foods removed from the diet included those with high glycemic indices (such as refined wheat products and refined sugars), common intolerances such as cow’s milk, and foods which negatively influence cardiovascular health such as hydrogenated oils. Participants also consumed daily supplements with established anti-inflammatory benefits. Omega-3 (Now Ultra omega-3) was taken in softgel form, containing 500 mg EPA and 250 mg DHA, at a dosage of 3 per day. Chlorella (Now chlorella) was taken in pill form, containing 1000 mg, at a dosage of 6 per day. Antioxidants (CanPrev antioxidant network) were taken in pill form, containing 100 mg coenzyme Q10, 200 mg *n*-acetyl-cysteine, 150 mg mixed tocopherols, 100 mg DL alpha lipoic acid, 60 mg green tea extract, 5.5 mg zinc, and 100 μg selenium, at dosage of 2 per day. Curcumin (AOR Inflanox) was taken in pill form, containing 400 mg, at a dosage of 3 per day. A vegetable-based protein powder (Progressive Vegessential) containing 27 g of protein was taken at a dosage of one scoop each morning.

Both the treatment group and control group were asked to complete a detailed diet record for 7 days at baseline, as well as 3 days at 1-month, 2-month, and 3-months in order to establish baseline eating habits and assess compliance throughout the intervention. Food intake was assessed using The Food Processor (ESHA Inc. 2014, version 10.14.2, Salem, OR). Compliance to the specific anti-inflammatory diet was also assessed by a detailed analysis of all diet records. Each food item was categorized as either a “food to consume,” a “food to avoid,” or a “neutral food” based on the parameter of the diet participants who were instructed to follow. Food was also categorized into servings in accordance with Canada’s Food Guide. Therefore, compliance score was based on standard servings of foods: subjects were instructed to eat vs. foods they were instructed to avoid. To account for differences in total energy intake, compliance scores were expressed as a ratio of the servings of foods to consume over the total servings of food (avoid + consume) multiplied by 100. The percent compliance was then generated. The treatment group then underwent an information seminar which explained the diet program followed by a one-on-one consultation with nutritionists whereby their diet records were reviewed in detail and necessary changes were discussed. Participants received information regarding foods to eat and avoid, a supplement intake schedule, and list of approved recipes. Participants in the treatment group received support via weekly phone calls from members of our research team as well as an online support group whereby participants could share recipes and experiences with one another. Participants in the control group were asked to maintain their current diets throughout the duration of the study.

### Measurement of serum inflammatory markers

Blood draws (20 ml) were taken from the antecubital vein of each participant at 1 pm at each of the three testing sessions (baseline, 1 month, and 3 months). Following extraction, the whole blood was allowed to clot for 30 min followed by centrifugation at 1000×*g* for 15 min. Serum was extracted and immediately stored at −80 °C until later analysis. Inflammatory mediators of interest included the proinflammatory cytokines IL-2, IL-1B, IL-6, TNF-alpha, IFN-y, acute phase protein CRP (C-reactive protein), eicosanoids PGE2 (prostaglandin E2), and LTB4 (leukotriene B4), as well as the anti-inflammatory cytokines IL-4, IL-10, and IL-1RA. Both pro- and anti-inflammatory cytokines were assessed due to findings that painful neuropathy has been shown to be associated with elevated levels of proinflammatory mediators and reduced levels of anti-inflammatory mediators while non-painful neuropathy has shown the opposite [12, 15]. Improvements in neuropathic pain may therefore relate to reductions in proinflammatory mediators and/or elevations in anti-inflammatory mediators. Analysis of pro- and anti-inflammatory cytokines was performed in triplicate via the Magpix Multiplex system and analyzed using Luminex software. CRP, PGE2, and LTB4 were analyzed in triplicate and quantified via enzyme-linked immunosorbent assay (R&D systems, Minneapolis, USA).

### Assessment of neuropathic pain

Participants were asked to complete the Neuropathic Pain Questionnaire (NPQ) at each of the three testing sessions, as a means of assessing self-reported neuropathic pain. The questionnaire consisted of 32 items pertaining to three unique categories including sensory items, affective items, and sensitivity items. Sensory items were those related to the specific type and severity of pain felt (e.g., degree of burning, stabbing, throbbing), affective items referred to those related to how the pain affected the participant in daily life (e.g., how irritating is your usual pain?) and sensitivity items related to how various stimuli may act to increase pain (e.g., increased pain due to heat). Participants were asked to rate their pain numerically on a scale from 0–100 whereby 0 indicated the complete absence of pain and 100 indicated the worst pain imaginable. Scores from each of the three categories were averaged for use in statistical analysis.

### Statistical analysis

Two-way (group × time) repeated measures ANOVA were performed to investigate possible changes in pain scores related to sensory and affective pain across three testing sessions (baseline, 1 month, 3 months). Two-way repeated measures ANOVA were also performed for the proinflammatory cytokine TNF-α and the eicosanoid PGE2. As the remaining inflammatory mediators as well as sensitivity pain scores were not normally distributed, non-parametric analyses were performed. A Friedman’s test of differences among repeated measures (baseline, 1 month, and 3 months) for the treatment group and control was performed. If the Friedman’s test resulted in a significant value, a Wilcoxon signed-rank test was then performed to provide specific information regarding which time points were significantly different from one another. Finally, A Mann-Whitney test was performed on change scores (3 months − baseline) between groups to establish if the change experienced significantly differed between groups. These data are expressed as means ± standard deviations. Correlations between changes in inflammatory mediators and neuropathic pain scores were assessed by means of Pearson’s r correlation. Statistical significance was set at *p* ≤ 0.05 for all tests. The proportional contribution of the change in each inflammatory mediator to the change in pain score was evaluated using a stepwise backward elimination multiple regression. Levels of F to enter and F to remove were set to correspond to p-levels of 0.05 and 0.10, respectively. In order to control for the potential influence of depression on scores of neuropathic pain, an additional multiple regression was performed while controlling for CES-D (center for epidemiological studies depression scale) scores of depression. Details of the CES-D have been previously described [14].

## Results

All participants from both the treatment and control group completed the entire 3-month duration of the study and were included in the analysis. No adverse events were reported. The participants’ overall compliance to the diet was assessed based on the average of the three diet records during the study (1 month, 2 months, and 3 months). One participant completed all three testing sessions but failed to produce the 2-month and the 3-month diet record. This participant had a dietary compliance over the first month of 92 %. All other participants completed each of the required diet records, and overall compliance ranged from 70 to 100 %, with a mean compliance of 89 %. A detailed analysis regarding specific diet adherence data will be presented elsewhere.

### Change in pain scores

Changes in sensory, affective, and sensitivity pain scores are shown in Table 2. There was a significant group × time interaction for the sensory component of the self-reported neuropathic pain scores (*p* < 0.01; Cohen’s *d* = 1.29). Post hoc analysis showed a significant reduction in sensory scores in the treatment group from both baseline to 1 month, as well as baseline to 3 months (*p* = 0.00 and *p* = 0.01, respectively). This included reductions of greater than 30 % for six participants and a reduction between 20 and 30 % for another three participants. Post hoc analysis showed a significant increase in sensory scores in the control group from baseline to 1 month (*p* = 0.04) but no significant change from baseline to 3 months (*p* = 0.210). No significant group × interaction was found for the affective component of the self-reported neuropathic pain scores (*p* = 0.17; Cohen’s *d* = 0.63). Of note, the change in affective neuropathic pain scores were significantly correlated to the previously reported scores related to changes in mood in this population [14]. In regard to the non-parametric analysis of sensitivity scores, the Mann-Whitney test indicated that change scores of sensitivity pain were not significantly different between the treatment group and control group (*U* = 36.0, *p* = 0.35). The Friedman test showed that there was no significant change in sensitivity pain scores for either the treatment group (*x*^2^ = 3.38, *p* = 0.19) or control group (*x*^2^ = 0.09, *p* = 0.96) across testing sessions.

**Table 2:** Changes in neuropathic pain questionnaire scores

	Treatment (*n* = 12)			Control (*n* = 8)			Two-way ANOVA (*p* value)	Mann-Whitney (*p* value)	Friedman [Treat.] (*p* value)
	Baseline	1 month	3 months	Baseline	1 month	3 months			
Sensory score	32.8 ± 23.4	23.4 ± 20.2**	19.8 ± 15.8**	18.1 ± 17.2	25.2 ± 22.2*	21.3 ± 20.1	<0.01	–	–
Affective score	34.7 ± 28.6	24.3 ± 21.9	21.2 ± 19.0	27.5 ± 24.4	20.1 ± 23.0	23.7 ± 25.3	0.18	–	–
Sensitivity score	26.8 ± 26.1	22.6 ± 21.2	22.6 ± 20.7	29.7 ± 32.9	33.7 ± 26.9	32.6 ± 26.3	–	0.35	0.19

All results are shown as mean ± SD. *p* values correspond to group × time interactions, Mann-Whitney change scores, and Friedman scores for treatment group, respectively (Friedman scores for control group not shown)

*Significantly different from baseline with *p* value <0.05

**Significantly different from baseline with *p* value <0.01

### Change in inflammatory mediators

Changes in serum levels of inflammatory mediators are shown in Table 3. When considering a proinflammatory composite score (average of IL-2, IL-6, IL-1β, TNF-α, and IFN-y), the Mann-Whitney test indicated that the change scores (3 months − baseline) were significantly different between the treatment group and the control group (*U* = 13.0, *p* = 0.01). The Friedman test showed that there was a statistically significant reduction in the proinflammatory composite scores in the treatment group (*x*^2^ = 6.50, *p* = 0.04), but no significant change in the control group (*x*^2^ = 5.25, *p* = 0.07). Post hoc analysis performed with the Wilcoxon signed-rank test showed significant reductions in the treatment group from both baseline to 1 month and baseline to 3 months (*z* = −2.197, *p* = 0.03; *z* = −2.275, *p* = 0.02 respectively). When analyzing each cytokine separately, the Mann-Whitney test indicated that the change scores (3 months − baseline) were significantly different between the treatment group and the control group for IFN-y (*U* = 13.0, *p* = 0.01.), IL-1β (*U* = 14.0, *p* = 0.01), and IL-2 (*U* = 12.0, *p* = 0.01) and showed a trend for CRP (*U* = 27.0, *p* = 0.10). The Friedman test showed that in the treatment group, there was a statistically significant reduction in IFN-y (*x*^2^ = 8.67, *p* = 0.01), IL-1β (*x*^2^ = 17.78, *p* < 0.01), IL-6 (*x*^2^ = 6.17, *p* < 0.05), and a trend for CRP (*x*^2^ = 4.5, *p* = 0.10). The Friedman test showed no statistically significant reductions for any inflammatory mediator in the control group. Post hoc analysis performed with the Wilcoxon signed-rank test showed significant reductions in the treatment group for IFN-y from baseline to 1 month and baseline to 3 months (*z* = −2.275, *p* = 0.02; *z* = −2.510, *p* = 0.01, respectively), as well as significant reductions in the treatment group for IL-1β from baseline to 1 month and baseline to 3 months (*z* = −3.059, *p* < 0.01; *z* = −2.934, *p* < 0.01, respectively), and a significant reduction in the treatment group for IL-6 from baseline to 1 month, and a trend from baseline to 3 months (*z* = −2.275, *p* = 0.02; *z* = −1.726, *p* = 0.08, respectively). Two-way repeated measures ANOVA were performed for the normally distributed mediator’s TNF-α and PGE2 and showed trends towards group × time interactions (*p* = 0.10; *p* = 0.07 respectively).

**Table 3:** Changes in inflammatory mediators

	Treatment (*n* = 12)			Control (*n* = 8)			Two-way ANOVA (*p* value)	Mann-Whitney (*p* value)	Friedman [Treat.] (*p* value)
	Baseline	1 month	3 months	Baseline	1 month	3 months			
Proinflammatory composite (pg/ml)	20.3 ± 34.5	13.1 ± 23.6*	14.6 ± 25.2*	9.8 ± 11.6	15.4 ± 22.3	15.7 ± 25.3	–	<0.01	0.04
CRP (ng/ml)	4474.7 ± 3578.9	3822.6 ± 3749.4	2865.0 ± 2684.9	2388.1 ± 2928.1	3074.0 ± 3026.4	2458.8 ± 3678.9	–	0.10	0.10
IL-2 (pg/ml)	21.3 ± 51.2	15.1 ± 41.7	17.2 ± 42.1	1.7 ± 3.4	2.9 ± 3.6	2.3 ± 3.3	–	<0.01	0.23
IL-6 (pg/ml)	13.9 ± 28.2	9.2 ± 21.3*	9.5 ± 19.3	9.0 ± 10.5	13.8 ± 21.2	13.5 ± 21.9	–	0.13	0.049
IL-1B (pg/ml)	0.9 ± 1.1	0.3 ± 0.3**	0.3 ± 0.2**	0.3 ± 0.3	0.4 ± 0.5	0.3 ± 0.2	–	<0.01	<0.01
TNF-α (pg/ml)	12.5 ± 3.6	11.8 ± 5.5	11.2 ± 4.1	9.8 ± 3.9	11.3 ± 6.7	12.9 ± 10.3	0.10	–	–
IFN-y (pg/ml)	52.9 ± 94.0	31.9 ± 57.5*	35.0 ± 68.4*	28.1 ± 46.8	48.8 ± 84.6	49.6 ± 95.3	–	<0.01	0.01
Anti-inflammatory Composite (pg/ml)	15.7 ± 13.7	17.2 ± 15.4	17.3 ± 19.1	28.8 ± 28.3	40.1 ± 44.9	36.3 ± 39.5	–	0.32	1.0
IL-4 (pg/ml)	7.5 ± 20.8	12.4 ± 23.9	16.2 ± 38.4	19.8 ± 37.2	37.4 ± 83.8	23.8 ± 46.3	–	0.54	0.63
IL-10 (pg/ml)	6.5 ± 12.9	11.2 ± 29.7	9.3 ± 22.0	5.9 ± 14.4	5.7 ± 13.7	6.3 ± 14.6	–	0.96	0.50
IL-1RA (pg/ml)	33.1 ± 26.2	27.8 ± 18.6	26.3 ± 16.0	60.6 ± 66.6	77.2 ± 77.2	78.8 ± 105.8	–	0.88	0.72
PGE2 (pg/ml)	496.5 ± 452.7	636.4 ± 544.9	353.0 ± 357.5	605.1 ± 491.2	605.6 ± 504.6	661.7 ± 503.7	0.07	–	–
LTB4 (pg/ml)	127.8 ± 181.8	119.8 ± 194.9	77.0 ± 73.8	121.3 ± 172.3	87.6 ± 59.3	145.1 ± 179.8	–	0.70	0.78

All results are shown as mean ± SD. *p* values correspond to group × time interactions, Mann-Whitney change scores, and Friedman scores for treatment group, respectively

Proinflammatory composite consists of a composite score averaging IL-2, IL-6, IL-1B, TNF-α, and IFN-y

Anti-inflammatory composite consists of a composite score averaging IL-4, IL-10, and IL1RA

*Significantly different from baseline with *p* value <0.05

**Significantly different from baseline with *p* value <0.01

Note: Adapted from “Targeting inflammation to influence mood following spinal cord injury: A randomized clinical trial” by David J. Allison and David S. Ditor, 2015, Journal of Neuroinflammation

### Relationship between inflammatory mediators and indices of pain

Results from the multiple regression are shown in Table 4. To help elucidate a potential mechanism between the reduction in neuropathic pain scores and inflammatory mediators, a stepwise backward multiple regression analysis was performed. When assessing the change in sensory pain score as the outcome variable, results from the regression analysis provided partial confirmation of the research hypothesis that change in sensory neuropathic pain is a function of the change in proinflammatory cytokines and eicosanoids. The three-variable model included the proinflammatory cytokines IL-2 and IFN-y, as well as the eicosanoid PGE2 (*R* = 0.689, *R*^2^ = 0.474). The overall *F* statistic for the model was 4.811, df = 3.16, *p* = 0.01. Standardized beta weights were −0.730 for IL-2, 0.544 for IFN-y, and 0.526 for PGE2. When assessing the change in affective pain score as the outcome variable, only PGE2 remained in the model (*R* = 0.558, *R*^2^ = 0.312). The overall *F* statistic for the one-variable model was 8.145, df = 1.18, *p* = 0.01, and the standardized beta weight was 0.558. Lastly, when assessing the change in sensitivity score as the outcome variable, a three-variable model including the proinflammatory cytokines IL-1B and IL-2 as well as the eicosanoid PGE2 remained (*R* = 0.715, *R*^2^ = 0.511). The overall *F* statistic for the three-variable model was 5.580, df = 3.16, *p* = 0.008. Standardized beta weights were 0.491 for IL-1B, −0.666 for IL-2, and 0.378 for PGE2.

**Table 4:** Backward stepwise multiple regression analysis of the relationship between the change in neuropathic pain scores and the change in inflammatory mediators

Outcome variable	Predictor variables	*b*	SE-b	Beta	*t*	*p* value	Tolerance	VIF
Sensory	IL-2	−1.386	.483	−0.73	−2.87	0.01	0.51	1.97
(*R* = 0.69, *R* ^2^ = 0.47)	IFN-y	.179	.081	0.54	2.21	0.04	0.52	1.91
	PGE2	.022	.008	0.53	2.79	0.01	0.92	1.08
Affective	PGE2	.02	.01	0.56	2.85	0.01		
(*R* = 0.56, *R* ^2^ = 0.31)								
Sensitivity	IL-1B	9.67	4.93	0.49	1.96	0.07	0.49	2.05
(*R* = 0.72, *R* ^2^ = 0.51)	IL-2	−1.49	.49	−0.67	−3.07	0.01	0.65	1.54
	PGE2	.02	.01	0.38	1.8	0.09	0.69	1.45

In order to account for the potential influence of depression on sensory neuropathic pain scores, an additional multiple regression analysis was performed while controlling for CES-D scores of depression. In this analysis, model 1 included CES-D scores of depression and model 2 included the inflammatory mediators IL-2, IFN-y, and PGE2. Model 1 resulted in a non-significant *R* square change score of 0.131 (*p* = 0.12) while model 2 resulted in a significant *R* square change score of 0.394 (*p* = 0.03). This suggests that the change in inflammatory mediators IL-2, IFN-y, and PGE2 explains an additional 39 % of the variability in the change in sensory neuropathic pain scores when the change in CES-D scores of depression has been statistically controlled for. When assessing standardized beta weights, CES-D scores were not significant (beta = 0.244, *p* = 0.23), while IL-2 and PGE2 remained significant (beta = −0.731, *p* = 0.01; beta = 0.495, *p* = 0.03, respectively), and IFN-y showed a trend towards significance (beta = 0.462, *p* = 0.07). The relationship between neuropathic pain scores and inflammatory mediators was also assessed at baseline by means of a stepwise backward elimination multiple regression analysis. When assessing baseline sensory neuropathic pain scores, IL-4 and PGE2 remained in the model (*R* = 0.672, *R*^2^ = 0.451). The overall *F* statistic for the model was 4.385, df = 3.16, *p* = 0.02. Standardized beta weights were −0.783 and 0.472 for IL-4 and PGE2, respectively. When assessing baseline affective neuropathic pain scores, IL-2, IFN-y, PGE2, and IL-4 remained in the model (*R* = 0.914, *R*^2^ = 0.836). The overall *F* statistic for the model was 14.295, df = 5.14, *p* < 0.01. Standardized beta weights were −0.580 for IL-2, 0.756 for IFN-y, 0.965 for PGE2, and −0.832 for IL-4. When assessing baseline sensitivity pain scores, IL-4 and LTB4 remained in the model (*R* = 0.909, *R*^2^ = 0.826). The overall *F* statistic for the model was 13.313, df = 5.14, *p* < 0.01. Standardized beta weights were −0.567 for IL-4 and 0.850 for LTB4.

## Discussion

The present study successfully obtained reductions in inflammatory mediators and scores of sensory neuropathic pain in individuals with chronic SCI by means of dietary alterations. The relationship between changes in inflammatory mediators and changes in sensory neuropathic pain scores was assessed via a stepwise, backward elimination multiple regression analysis. It was demonstrated that approximately 47 % of the change in sensory neuropathic pain scores could be explained by the change in three inflammatory mediators including IL-2, IFN-y, and PGE2. Both IFN-y and PGE2 demonstrated positive relationships whereby a one-unit decrease in these mediators was related to a respective 0.544- and 0.526-unit decrease in sensory neuropathic pain scores. IL-2 demonstrated a negative relationship whereby a one-unit increase was related to a 0.730-unit decrease in sensory neuropathic pain scores. As changes in depression may also be expected to influence changes in neuropathic pain, an additional multiple regression analysis while controlling for CES-D scores of depression was conducted. This analysis showed that the inflammatory mediators IL-2, IFN-y, and PGE2 explained an additional 39 % of the change in sensory neuropathic pain scores even when controlling for the change in CES-D scores. The relationship between these inflammatory mediators and sensory neuropathic pain scores may be explained by the various peripheral and central mechanisms through which these mediators have been shown influence pain.

Under non-pathological conditions, free nerve endings in the periphery detect painful mechanical, thermal, or chemical stimuli and generate nerve impulses which then travel along afferent A-delta, or C fibers to the dorsal horn of the spinal cord. At this point, A-delta fibers synapse with second order neurons (while C fibers first synapse with interneurons) before ascending along the spinothalamic tract to the thalamus and somatosensory cortex whereby the magnitude and location of pain is processed. Under pathological conditions, chronic inflammation may influence pain signaling at different points along this pathway.

PGE2 has been shown to be a key factor in inflammatory-evoked pain by means of inducing sensitization of peripheral nociceptors as well as by inducing central changes concerning the processing of spinal nociceptive input [16]. Peripherally, PGE2 acts on corresponding receptors (EP) on the nociceptor which causes a protein kinase A (PKA)-mediated phosphorylation of sodium channels thereby causing peripheral sensitization [16]. PGE2 has also been shown to induce similar effects centrally by inducing membrane depolarization of dorsal horn neurons leading to action potential generation [17]. There is also evidence that PGE2 may target the inhibitory glycine receptor thereby reducing inhibitory glycinergic synaptic transmission. This provides a second mechanism by which PGE2 may facilitate transmission of nociceptive input through the dorsal horn of the spinal cord [18].

There is also evidence that LTB4 may act centrally to contribute to neuropathic pain. LTB4 can activate BLT1 (leukotriene B4 receptor 1) receptors, which are expressed on the membrane of spinal dorsal horn neurons during neuropathic pain [19]. This activation induces the enhancement of NMDA currents via intracellular G-proteins. The enhancement of NMDA receptor sensitivity of dorsal horns may then contribute to central sensitization and pain hypersensitivity [19].

IFN-y has also been shown to induce central effects which contribute to the enhancement of neuropathic pain. Spinal microglia express receptors for IFN-y. Once activated by IFN-y, they have been shown to induce the production of bioactive factors such as cytokines and neurotrophic factors [20, 21] thereby influencing the excitability of the dorsal horn pathway [22] and injury-induced pain behaviors [23]. Through this process, IFN-y is able to indirectly enhance pain processing in the dorsal horn and influence neuropathic pain.

The negative relationship found between IL-2 and sensory pain scores can be explained by previous reports which have demonstrated antinociceptive properties of IL-2. Pain transmission can be depressed by means of inducing hyperpolarization, thereby reducing neuronal excitability, and depressing the release of nociceptive neurotransmitters [24]. Free intracellular calcium (Ca^2+^) plays a key role in the release of nociceptive neurotransmitters such as substance P from the presynaptic neuron [25, 26]. It is through this mechanism that opioids have been demonstrated to produce their analgesic effects. The activation of opioid receptors has been shown to suppress high threshold Ca^2+^ currents in rat dorsal root ganglion thereby inducing presynaptic inhibition [27, 28]. IL-2 administration has been demonstrated to induce a similar influence on high threshold Ca^2+^ currents and inhibit the depolarization-evoked increase in intracellular Ca^2+^ concentration [29]. The fact that the administration of the u-opioid antagonist naloxone produces dramatic reductions in this effect suggests that IL-2 may also be producing these effects by acting on opioid receptors [30].

Although speculative, the mechanisms discussed above may explain the link between the dietary-induced reduction in inflammation and the reductions in neuropathic pain scores observed in the current study. The fact that dietary alterations may target the underlying mechanisms of neuropathic pain may explain its effectiveness as a treatment modality. This could provide some advantage over traditional pharmacological treatments which focus on symptom relief aimed at downstream targets such as those involving the direct reduction of neuronal hyperexcitability. Such strategies have demonstrated mixed results in terms of efficacy, universality, and associated side effects. Examples include the use of tricyclic antidepressants which have shown efficacy for central pain [31], but also a lack of effect for HIV neuropathy [32, 33], or chemotherapy-induced neuropathic symptoms [34, 35]. SSRI’s have also been shown to produce only weak analgesic effects (Sindrup et al., [36]; Sindrup et al., [37]; Otto et al., [38]). The use of cannabinoids has been shown to relieve peripheral neuropathic pain in some studies (Karst M et al., [39]; Nurmikko et al., [40]; Baron et al., [41]) but has shown no effect in painful poly-neuropathy [42]. Anticonvulsants such as gabapentin have been shown to be effective for the relief of painful polyneuropathy [43] but have also demonstrated a lack of effect in several studies [31, 44–46]. Finally, opioids have been demonstrated, fairly consistently to benefit symptoms of neuropathic pain [47–51]; however, the risk of addiction and gastrointestinal side effects may make long-term use inappropriate [52]. The fact that the etiological basis of neuropathic pain may be highly variable from one individual to another makes it difficult to establish a universally effective treatment and may explain the marginal effectiveness of many drug trials. While a particular drug treatment may demonstrate a large effect on a small subgroup of participants, it also commonly shows a complete lack of effect in others, resulting in minimal overall efficacy. As dietary alterations are capable of targeting the underlying inflammatory mechanisms of neuropathic pain, they may provide a more widely applicable treatment option.

In addition to the potential for a greater universality and reduction in undesirable side-effects, anti-inflammatory strategies such as that utilized in the current study, may provide benefits comparable in magnitude to traditional pharmacological methods. Gabapentin is currently among the most promising pharmaceutical treatments for neuropathic pain. Studies which examined an 8-week administration of gabapentin have demonstrated reductions in neuropathic pain scores of 40.6 % in individuals with painful diabetic neuropathy [53], 33.3 % in individuals with postherpetic neuralgia [54], and 21.1 % in individuals with mixed neuropathic pain syndromes [55] (based on an 11-point Likert scale). The 39.6 % reduction in sensory neuropathic pain scores achieved in the current study is therefore of comparable or better magnitude to that achieved following gabapentin administration. Further, according to Farrar et al. [56], a 30 % reduction in a pain intensity numerical rating scale (PI-NRS) represents a clinically important difference in pain [56].

It has also been suggested that the TH1:TH2 balance may play a role in the severity of neuropathic pain. Studies related to painful neuropathy following SCI have shown elevated levels of the proinflammatory cytokines TNF, IL-6, and IL-2, combined with reduced concentrations of the anti-inflammatory cytokines IL-10 and IL-4 [12, 57]. Alternatively, individuals with painless neuropathies have been shown to have elevated levels of anti-inflammatory cytokines [15]. It may therefore be possible that improvements in neuropathic pain in the current study would have been even more substantial had anti-inflammatory cytokines been successfully increased.

Several potential study limitations should be noted. First, the study was only single blinded. While the examiner was blinded to group allocation during all blood analysis, participants were aware of their group assignment. Although placebo supplements could have been provided to the control group, it was not possible to adequately blind participants to all aspects of the diet. The treatment group underwent a highly restrictive diet devoid of any refined, processed, or fried foods while the control group was free to consume such foods, making distinction between groups quite obvious. For this reason, the potential for a placebo effect to have influenced the changes in neuropathic pain to some extent cannot be ignored. A recent review by Cragg et al. [58] concluded that on average, a weak yet statistically significant placebo response was apparent in individuals with central neuropathic pain [58]. Second, it is not possible to elucidate the specific mechanisms related to the reductions in inflammation, nor is it possible to discern which aspects of the dietary intervention may have had the strongest effects. It will be necessary for future studies to examine aspects such as transcription factor activity and membrane composition in order to truly elucidate the means by which such interventions act to reduce inflammation and improve symptoms of neuropathic pain. Third, although our sample was quite representative of the SCI population in Canada, in terms of age, level, and severity of injury [59], the results are not necessarily generalizable to other chronic inflammatory populations or all forms of neuropathic pain. It is also worth noting that the use of interventions which target inflammation by such means as diet or exercise requires commitment to major lifestyle alterations and it may be possible that improvements are less drastic and occur at a slower rate than that of certain pharmaceuticals. However, given the potential for such anti-inflammatory interventions to provide a more widely applicable aid for neuropathic pain symptoms (in addition to contributing to a multitude of other health benefits), while lacking the side effects associated with traditional pain medications, there is seemingly little reason not to promote such lifestyle alterations as a treatment option.

## Conclusions

The present study demonstrated that it was possible to improve symptoms of neuropathic pain in spinal cord injury by means by reducing levels of inflammation. Secondarily, appropriate dietary alterations may be one such intervention strategy which could be used to reduce inflammation and induce such benefits. This influence is worthy of further examination as it may help to reduce the reliance on traditional pain medications and provide a safe, sustainable, and more widely applicable treatment modality.

## Abbreviations

Ca^2+^, calcium; CRP, C-reactive protein; IFN-y, interferon gamma; IL, interleukin; LTB4, leukotriene B4; NPQ, neuropathic pain questionnaire; PGE2, prostaglandin E2; SCI, spinal cord injury; SSRI, selective serotonin reuptake inhibitor; TNF-α, tumor necrosis factor alpha

## Additional files

Additional file 1: CONSORT 2010 Flow Diagram. (DOC 52 kb) Additional file 2: Raw Data. (DOCX 56 kb)
