# Supplementary material for: Targeting inflammation as a treatment modality for neuropathic pain in spinal cord injury: a randomized clinical trial
Source: J Neuroinflammation. 2016 Jun 17;13:152. doi: 10.1186/s12974-016-0625-4 (PMC4912827; doi:10.1186/s12974-016-0625-4)
Supplement: Additional file 1: — CONSORT 2010 Flow Diagram. (DOC 52 kb) [file 12974_2016_625_MOESM1_ESM.doc]

**
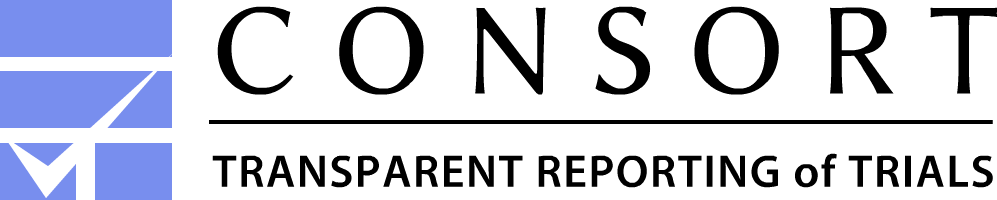
**

**CONSORT 2010 Flow Diagram**

**Allocation**

**Analysis**

**Follow-Up**

**Enrollment**

Assessed for eligibility (n= 24 )

Excluded (n= 4 )

♦  Not meeting inclusion criteria (n=1 )

♦  Declined to participate (n=3 )

♦  Other reasons (n=0 )

Analysed (n=12 )
♦ Excluded from analysis (give reasons) (n=0 )

Lost to follow-up (give reasons) (n=0 )

Discontinued intervention (give reasons) (n=0 )

Allocated to intervention (n=12 )

♦ Received allocated intervention (n=12 )

♦ Did not receive allocated intervention (give reasons) (n=0 )

Lost to follow-up (give reasons) (n=0 )

Discontinued intervention (give reasons) (n=0)

Allocated to intervention(control) (n= 8 )

♦ Received allocated intervention (n= 8 )

♦ Did not receive allocated intervention (give reasons) (n=0 )

Analysed (n=8 )
♦ Excluded from analysis (give reasons) (n=0 )

Randomized (n= 20 )
