# Supplementary material for: Targeting inflammation as a treatment modality for neuropathic pain in spinal cord injury: a randomized clinical trial
Source: J Neuroinflammation. 2016 Jun 17;13:152. doi: 10.1186/s12974-016-0625-4 (PMC4912827; doi:10.1186/s12974-016-0625-4)
Supplement: Additional file 2: — Raw Data. (DOCX 56 kb) [file 12974_2016_625_MOESM2_ESM.docx]

**Raw Data**
